# Identification of Core Biomarkers Associated with Outcome in Glioma: Evidence from Bioinformatics Analysis

**DOI:** 10.1155/2018/3215958

**Published:** 2018-10-10

**Authors:** Rong-Xin Geng, Ning Li, Yang Xu, Jun-hui Liu, Fan-en Yuan, Qian Sun, Bao-Hui Liu, Qian-Xue Chen

**Affiliations:** ^1^Department of Neurosurgery, Renmin Hospital of Wuhan University, Wuhan, China; ^2^Department of Cardiology, Renmin Hospital of Wuhan University, Wuhan, China

## Abstract

Glioma is the most common neoplasm of the central nervous system (CNS); the progression and outcomes of which are affected by a complicated network of genes and pathways. We chose a gene expression profile of GSE66354 from GEO database to search core biomarkers during the occurrence and development of glioma. A total of 149 samples, involving 136 glioma and 13 normal brain tissues, were enrolled in this article. 1980 differentially expressed genes (DEGs) including 697 upregulated genes and 1283 downregulated genes between glioma patients and healthy individuals were selected using GeoDiver and GEO2R tool. Then, gene ontology (GO) analysis as well as Kyoto Encyclopedia of Genes and Genomes (KEGG) pathway analysis were carried out using the Database for Annotation, Visualization and Integrated Discovery (DAVID). Moreover, Cytoscape with Search Tool for the Retrieval of Interacting Genes (STRING) and Molecular Complex Detection (MCODE) plug-in was employed to imagine protein-protein interaction (PPI) of these DEGs. The upregulated genes were enriched in cell cycle, ECM-receptor interaction, and p53 signaling pathway, while the downregulated genes were enriched in retrograde endocannabinoid signaling, glutamatergic synapse, morphine addiction, GABAergic synapse, and calcium signaling pathway. Subsequently, 4 typical modules were discovered by the PPI network utilizing MCODE software. Besides, 15 hub genes were chosen according to the degree of connectivity, including TP53, CDK1, CCNB1, and CCNB2, the Kaplan-Meier analysis of which was further identified. In conclusion, this bioinformatics analysis indicated that DEGs and core genes, such as TP53, might influence the development of glioma, especially in tumor proliferation, which were expected to be promising biomarkers for diagnosis and treatment of glioma.

## 1. Introduction

Gliomas comprise about 30% of all brain tumors and central nervous system (CNS) tumors and 80% of all malignant brain tumors [1]. Malignant glioma is the most common brain glioma (accounting for ~70%) with an annual incidence of 5 per 100,000 and an extremely malignant clinical outcome. Taking the most common type, glioblastoma (GBM), for example, possesses a median overall survival of only 10–15 months, which gives rise to serve health burden [2]. The pathogenesis of gliomas is very complicated; although a great many genes and proteins take part in the occurrence and development of gliomas, the mechanism still remains not clear [3]. At present, genetic factors and residual embryonic primitive cells in the brain are vital endogenous influences contributing to glioma while harmful physical and chemical factors along with virus infection serve as exogenous factors [4, 5]. Regardless of these different factors of pathogenesis, gliomas share similar mechanisms of oncogenesis, such as retinoblastoma (RB), p53, and receptor tyrosine kinase (RTK) signaling pathways, which play critical parts in the development of glioma, especially in GBM. Hence, genes and proteins that can modulate these pathways are not only used for therapeutic targets and diagnostic markers but can also be utilized to assess the pathological characteristics of gliomas [6, 7]. For example, isocitrate dehydrogenase (IDH) [8], epidermal growth factor receptor (EGFR) [9], and neurofibromatosis type 1 (NF1) [10] have been the center of attention in glioma genesis.

Currently, the diagnosis of gliomas mainly depends on pathological feature and medical imaging such as CT, MRI, DSA, PET, and SPECT, which have been closely related to experience of surgeons [11]. Because of lacking specificity of auxiliary examination biomarker, it is difficult for surgeons to obtain an accurate diagnosis and treatment of glioma as early as possible, so that many patients miss the optimal chance for surgery, thus increasing the risk of death. On the other hand, the common treatment strategies of gliomas consist of surgical resection as well as chemo and radiotherapy while targeting drugs are still limited [12]. Hence, searching for specific and sensitive biomarkers as well as some core genes or proteins as therapeutic target will benefit the diagnosis and treatment of gliomas.

At present, high-throughput sequencing has been applied as a very critical tool for medical research [13]. In this analysis, we chose GSE66354 from Gene Expression Omnibus (GEO) and used GeoDiver and GEO2R online tool to find the differentially expressed genes (DEGs). Subsequently, we made PPI network of the DEGs and selected core genes with a high degree of connectivity. In addition, analysis of biological process (BP), molecular function (MF), cellular component (CC), and KEGG pathways of the DEGs and four modules was performed. Moreover, overall survival (OS) analysis of these core genes was carried out. Then, the correlation analysis using TCGA database was employed to observe the potential relationship between genes. Briefly, this study would provide novel targets for diagnosis and treatment of glioma.

## 2. Results

### 2.1. Identification of DEGs and Hub Genes

**Figure 1:**
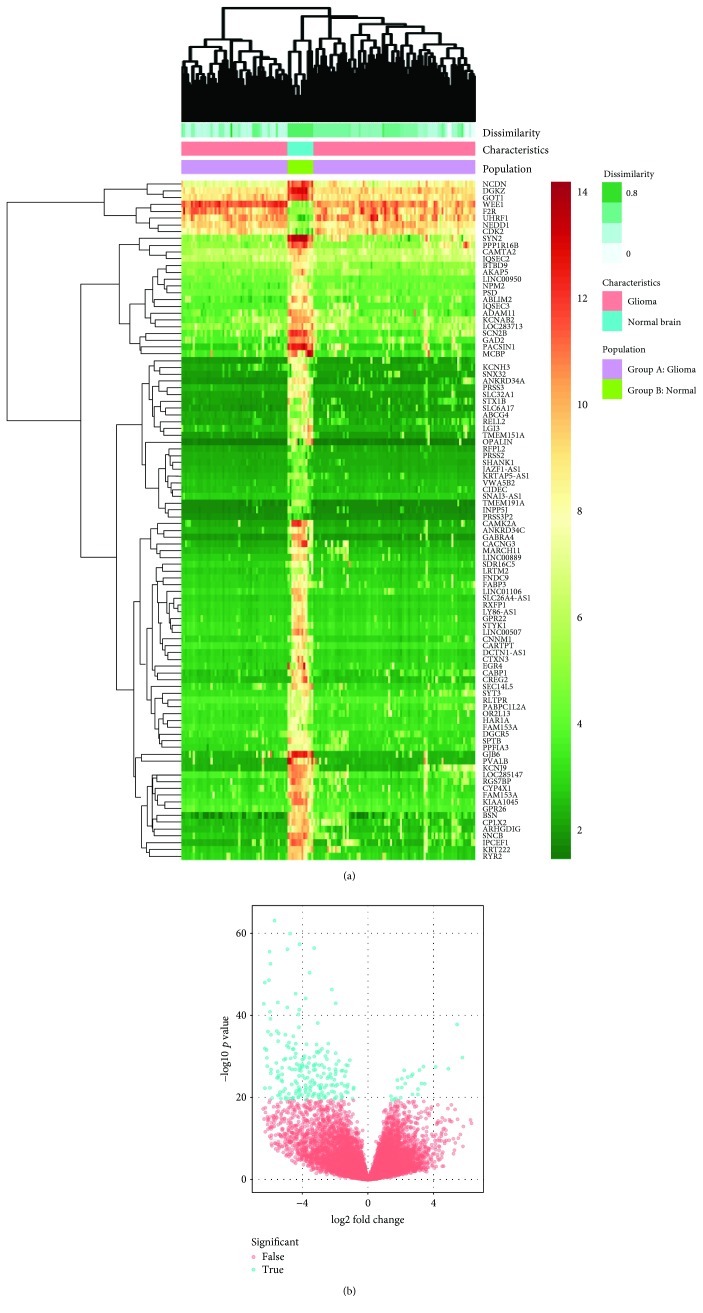
(a) Differentially expressed gene expression heat map of glioma (top 100 upregulated and downregulated genes). (b) Differentially expressed genes were selected by volcano plot filtering (fold change ≥ 1 and *p* value ≤ 0.05). The blue point in the plot represents the differentially expressed genes with statistical significance.

There were 136 glioma (including 21 high-grade gliomas, 15 pilocytic astrocytomas, 19 medulloblastomas, 17 atypical teratoid/rhabdoid tumors, and 64 ependymomas) samples and 13 normal samples in our study. The results of DEGs analysis were freely available in GeoDiver (https://www.geodiver.co.uk/). The heat map and volcano plot showed these DEGs in glioma (Figure 1). The GEO2R online analysis tool was applied to detect the DEGs, using adjust *p* value < 0.01 and ∣logFC∣ ≥ 2 as cutoff criteria. A total of 1980 DEGs were detected after the analysis of GSE66354; of which, 697 were upregulated genes and 1283 were downregulated. Besides, 15 hub genes with a high degree of connectivity were selected (Table 1).

### 2.2. GO Function and KEGG Pathway Analysis of DEGs

**Figure 2:**
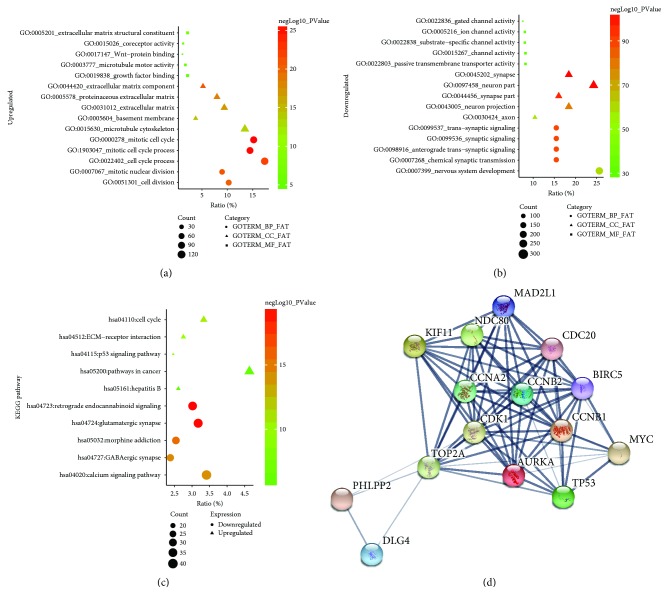
GO analysis results showed that upregulated DEGs (a) and downregulated DEGs (b) were particularly enriched in BP, MF, and CC. (c) The most significantly enriched KEGG pathway of the upregulated and downregulated DEGs. (d) The protein-protein interaction network of top 15 hub genes. GO: gene ontology; BP: biological process; MF: molecular function; CC: cell component; KEGG: Kyoto Encyclopedia of Genes and Genomes.

To obtain a more in-depth understanding of the selected DEGs, GO function and KEGG pathway analysis were applied using DAVID (Figure 2). GO analysis results showed that upregulated DEGs and downregulated DEGs were particularly enriched in biological process (BP), including mitotic cell cycle, mitotic cell cycle process, cell cycle process, mitotic nuclear division, and cell division for upregulated DEGs, and for downregulated DEGs including synaptic signaling, anterograde trans-synaptic signaling, trans-synaptic signaling, chemical synaptic transmission, and nervous system development (Table 2). For molecular function (MF), the upregulated DEGs were mainly enriched in extracellular matrix structural constituent, coreceptor activity, Wnt protein binding, microtubule motor activity, and growth factor binding, and the downregulated DEGs were enriched in gated channel activity, ion channel activity, substrate-specific channel activity, channel activity, and passive transmembrane transporter activity (Table 2). In addition, GO cell component (CC) analysis also displayed that the upregulated DEGs were significantly enriched in the extracellular matrix component, proteinaceous extracellular matrix, extracellular matrix, basement membrane, and microtubule cytoskeleton, and downregulated DEGs were enriched in synapse, neuron part, synapse part, neuron projection, and axon (Table 2).

Figure 2(c) and Table 3 show the most significantly enriched KEGG pathway of the upregulated and downregulated DEGs. The upregulated DEGs were enriched in cell cycle, ECM-receptor interaction, p53 signaling pathway, pathways in cancer, and hepatitis B, while the downregulated DEGs were enriched in retrograde endocannabinoid signaling, glutamatergic synapse, morphine addiction, GABAergic synapse, and calcium signaling pathway.

### 2.3. The Kaplan-Meier Plotter and Expression Level of Hub Genes

**Figure 3:**
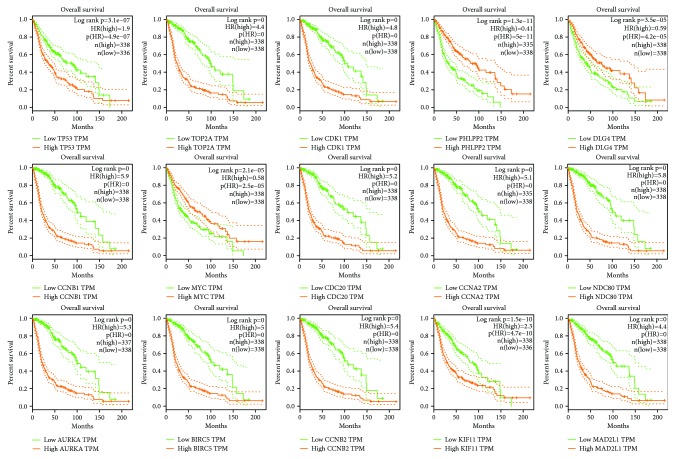
Prognostic value of 15 genes (TP53, TOP2A, CDK1, PHLPP2, DLG4, CCNB1, MYC, CDC20, CCNA2, NDC80, AURKA, BIRC5, CCNB2, KIF11, and MAD2L1) in glioma patients. HR: hazard ratio.

**Figure 4:**
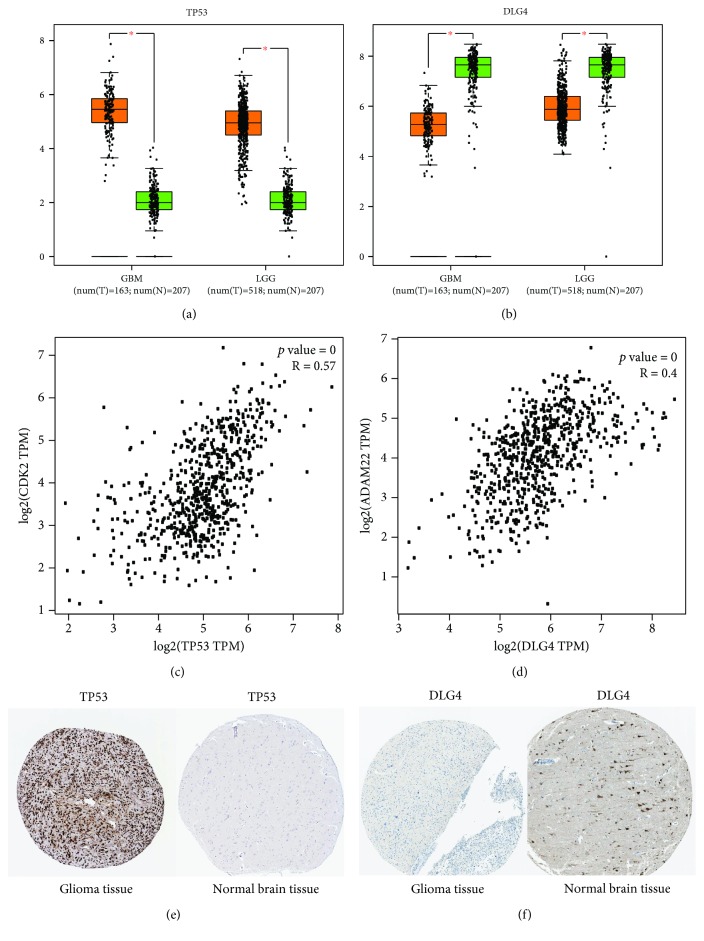
(a) Expression level of TP53 in cancer and normal tissues. GBM: glioblastoma multiforme; LGG: brain lower grade glioma; ^∗^*p* < 0.05. (b) Expression level of DLG4 in cancer and normal tissues; ^∗^*p* < 0.05. (c) The correlation analysis between TP53 and CDK2. TP53 and CDK2 are obviously positively correlated. (d) The correlation analysis between DLG4 and ADAM22. DLG4 and ADAM22 are obviously positively correlated. (e) TP53 protein was strongly upregulated in glioma tissues compared with normal brain tissues based on the Human Protein Atlas database. The normal brain tissue of TP53 was from a male, age 62, (patient ID: 1609; staining: not detected; intensity: negative; quantity: negative; location: none), and the glioma tissue was from a male, age 61, (patient ID: 2522; staining: high; intensity: strong; quantity: >75%; location: nuclear). (f) DLG4 protein was strongly downregulated in glioma tissues compared with normal brain tissues based on the Human Protein Atlas database. The normal brain tissue of DLG4 was from a male, age 45, (patient ID: 2521; staining: medium; intensity: moderate; quantity: >75%; location: membranous nuclear), and the glioma tissue was from a male, age 48 (patient ID: 3092; staining: not detected; intensity: negative; quantity: negative; location: none).

The prognostic information of the 15 hub genes was freely available at http://gepia.cancer-pku.cn/detail.php. It was found that expression of TP53 (HR 1.9, *p* = 3.1 × 10^–7^) was associated with worse overall survival (OS) for glioma patients, as well as TOP2A (HR 4.4, *p* = 0), CDK1 (HR 4.8, *p* = 0), CCNB1 (HR 5.9, *p* = 0), CDC20 (HR 5.2, *p* = 0), CCNA2 (HR 5.1, *p* = 0), NDC80 (HR 5.8, *p* = 0), AURKA (HR 5.3, *p* = 0), BIRC5 (HR 5, *p* = 0), CCNB2 (HR 5.4, *p* = 0), KIF11 (HR 2.3, *p* = 1.5 × 10^–10^), and MAD2L1 (HR 4.4, *p* = 0), while expression of PHLPP2 (HR 0.41, *p* = 1.3 × 10^–11^), DLG4 (HR 0.59, *p* = 3.5 × 10^–5^), and MYC (HR 0.58, *p* = 2.1 × 10^–5^) was associated with better overall survival (OS) for glioma patients (Figure 3). Then, we used GEPIA to detect the hub gene expression level between cancer and normal brain tissue, and Figures 4(a) and 4(b) reflect that compared to normal brain tissue, the expression level of TP53 significantly increased in cancer tissue while the expression level of DLG4 significantly decreased in glioma. The immunohistochemical data of patients with or without glioma based on the Human Protein Atlas (HPA) also verified the expression of these hub genes (Figures 4(e) and 4(f)).

### 2.4. Hub Genes and Module Screening from PPI Network

Based on the information in the STRING protein query from public databases, we made the PPI network of the top 15 hub genes with higher degree of connectivity (Figure 2(d)). We selected TP53, TOP2A, CDK1, CCNB1, CDC20, CCNA2, NDC80, AURKA, BIRC5, CCNB2, KIF11, and MAD2L1, which with worse overall survival situation according to the Kaplan-Meier plotter. Based on the GO function, the KEGG pathway analysis, and the survival analysis, we found that TP53, CDK1, CCNB1, and CCNB2 were enriched in cell cycle, especially in p53 signaling pathway.

**Figure 5:**
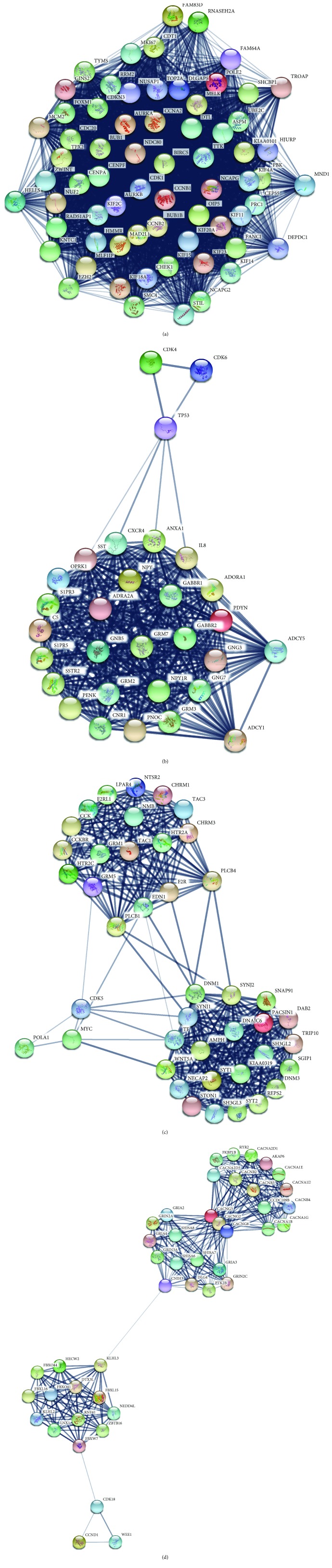
Top 4 modules from the protein-protein interaction network. (a) Module 1: score = 63.788, (b) module 2: score = 24.69, (c) module 3: score = 18.537, and (d) module 4: score = 14.186.

In order to detect significant modules in this PPI network, we used MCODE plug-in. The top 4 modules were selected (Figure 5). KEGG pathway enrichment analysis showed that these 4 modules were mainly correlated with cell cycle, neuroactive ligand-receptor interaction, calcium signaling pathway, and arrhythmogenic right ventricular cardiomyopathy (ARVC) (Table 4).

## 3. Discussion

The rising trend of the glioma morbidity has raised our attention in recent years, which is mainly caused by the failure to early diagnosis and treatment. Therefore, sensitive and specific biomarkers as well as core therapeutic targets of glioma are urgently needed to be screened. In the present analysis, 136 glioma samples (including 21 high-grade gliomas, 15 pilocytic astrocytomas, 19 medulloblastomas, 17 atypical teratoid/rhabdoid tumors, and 64 ependymomas) and 13 normal samples were enrolled from the GEO database of GSE66354. A total of 1980 DEGs were identified, including 697 upregulated genes and 1283 downregulated genes. To obtain an in-depth understanding of these DEGs, we performed the GO function and KEGG pathway analysis of these DEGs. And we found that the upregulated DEGs are mainly implicated with cell cycle, ECM-receptor interaction, and p53 signaling pathway, while the downregulated genes were enriched in retrograde endocannabinoid signaling, glutamatergic synapse, morphine addiction, GABAergic synapse, and calcium signaling pathway. In the meantime, we verified the effects of these hub genes on survival in patients with glioma. Of these 15 hub genes, PHLPP2, DLG4, and MYC displayed significant positive correlation with overall survival while TP53, TOP2A, CDK1, CCNB1, CDC20, CCNA2, NDC80, AURKA, BIRC5, CCNB2, KIF11, and MAD2L1 negatively correlated with overall survival in patients with glioma. Subsequently, we further confirmed the expression of TP53 and DLG4 in GBM and LGG.

### 3.1. DEGs Are Promising Candidates for the Diagnosis of Glioma

Our analysis picked up 1980 DEGs between patients with glioma and normal individuals. In our heat map, a total of 100 DEGs with most differential expression were captured. We hypothesized that these DEGs would be promised to be candidates for the diagnosis of glioma in future. In fact, some of these DEGs have been already uncovered to be good predictors of glioma. For instance, WEE1 is a regulator of the G2 checkpoint in cell. The WEE1-positive nuclear area is correlated with malignancy grade and WEE1 is associated with prognosis in GBM inversely [14]. LGI3, a secreted protein member of leucine-rich glioma inactivated (LGI) family, is predominantly expressed in the brain, skin, and adipose tissues, exerting roles as a multifunctional cytokine [15]. Studies have revealed that low expression levels of LGI3 are obviously associated with poor prognosis of glioma [16]. Collectively, the clinical value of these DEGs needs to be further explored.

### 3.2. Hub Genes May Serve as Core Therapeutic Targets in Glioma

In this study, we selected 15 hub genes in glioma, which were in the core nodes in PPI network; thus, they might be the key therapeutic targets to combat glioma. TOP2A, one of the important molecular markers, would predict response to chemotherapy. In glioma, high levels of TOP2A mRNA have been noted in GBM in comparison with grade II and III astrocytomas and also correlate with tumor TOP2A protein levels. Interestingly, temozolomide inhibited TOP2A activity and siRNA knocked down of TOP2A rendered a glioma cell line resist. Hence, the transcript of TOP2A should be a good prognostic indicator in GBM patients receiving temozolomide chemotherapy. AURKA is located on chromosome 20q13, frequently amplified and overexpressed in human malignancies, involving breast cancer [17], pancreatic cancer [18], and gastric cancer [19]. A recent study has demonstrated that AURKA could confer self-renewal capacity of competing away the binding of AXIN from *β*-catenin, inducing *β*-catenin stabilization and activating Wnt signaling in glioma-initiating cells [20]. Hence, endogenous or exogenous drugs that could target these genes will combat glioma. For example, miR-124, an important downstream target gene of hedgehog (Hh) signaling, potentially interacts with the 3′-UTR region of AURKA; thus, upregulating miR-124 significantly reduces the expression of AURKA and inhibits the proliferation and growth of human glioma cells [21].

### 3.3. TP53 Is Essential for the Progression of Glioma

Tumor protein p53 (TP53), also known as BCC7, LFS1, P53, and TRP53, encodes a tumor suppressor protein containing transcriptional activation, DNA binding, and oligomerization domains [22]. The encoded protein responds to diverse cellular stresses to regulate expression of target genes, thereby inducing cell cycle arrest, apoptosis, senescence, DNA repair, or changes in metabolism [23]. Mutations in this gene are associated with a variety of human cancers, such as breast cancer [24], prostate cancer [25], liver cancer [26], and colorectal carcinoma [27]. The PPI network analysis found that TP53 had a higher combined score with CDK2.  Figure 4(c) shows the results of the correlation analysis between TP53 and CDK2. TP53 and CDK2 were obviously positively correlated. The CDK2 gene encodes a member of the serine/threonine protein kinase family that is involved in cell cycle regulation [28]. The CDK2 protein is implicated in the control of cell cycle progression [29]. Thus, targeting of cell cycle checkpoints in cancer cells may inhibit tumor growth and induce cell death. CDK2 is a vital regulator of S-phase progression and has been regarded as an anticancer drug target in several studies [30, 31]. Recent studies show that for both DNA damage- and oncogene-induced cellular senescence, CDK2 transcript and protein are inhibited in a p53- and RB-dependent manner, and this repression is necessary for cell cycle exit during senescence, suggesting that there may exist a potential relationship between TP53 and CDK2 [32]. Limited by few studies about the relationship between TP53 and CDK2 in glioma, further research is necessary to clarify the underlying correlation and mechanism between TP53 and CDK2.

Cyclin-dependent kinase 1 (CDK1), also called CDC2, CDC28A, and P34CDC2, encodes the protein of a member of the serine/threonine protein kinase family. This protein is a catalytic subunit of the highly conserved protein kinase complex known as M-phase promoting factor (MPF), which is essential for G1/S- and G2/M-phase transitions of eukaryotic cell cycle [33]. Mitotic cyclins stably associate with this protein and function as regulatory subunits. The kinase activity of this protein is controlled by cyclin accumulation and destruction through the cell cycle. The phosphorylation and dephosphorylation of this protein also play important regulatory roles in cell cycle control [34]. A previous study indicates that apoptosis induced by cdk1 inhibition is dependent on caspase activation and is concomitant with upregulation of transcriptional targets of TP53 [35]. Since CDK1 is associated with many types of human cancers, it is believed that CDK1 might play an important role in diagnosis and therapy of glioma.

Taken together, our bioinformatics analysis identified DEGs and they might play central roles in the occurrence, development, and prognosis of glioma. In this study, a total of 1980 DEGs and 15 hub genes were selected, and TP53, CDK1, CCNB1, and CCNB2 might be the core genes of gastric cancer. In order to get more accurate correlation results, we need to start a series of verification experiments later to confirm the results of this prediction. Anyway, this study could provide some powerful evidence for the future genomic individualized treatment of glioma.

## 4. Materials and Methods

### 4.1. Microarray Data

We chose a gene expression profile of GSE66354 from GEO database. GSE66354 was based on the Agilent GPL570 platform ([HG-U133_Plus_2] Affymetrix Human Genome U133 Plus 2.0 Array). The GSE66354 dataset included 149 samples, containing 136 glioma samples (including 21 high-grade gliomas, 15 pilocytic astrocytomsa, 19 medulloblastomas, 17 atypical teratoid/rhabdoid tumors, and 64 ependymomas) and 13 normal brain tissues [36]. Besides, we downloaded the Series Matrix File of GSE66354 from GEO database.

### 4.2. Differential Gene Expression Analysis (DGEA)

GeoDiver (https://www.geodiver.co.uk/) was an online web application for performing differential gene expression analysis (DGEA) and generally applicable gene-set enrichment analysis (GAGE) on gene expression datasets from the publicly available Gene Expression Omnibus (GEO) [37]. GeoDiver used the limma R package to identify differentially expressed genes by fitting a linear model to each gene, which estimated the fold change in expression while accounting for standard errors by applying empirical Bayes smoothing. The adjust *p* values were used to decrease the false positive rate utilizing Benjamini and Hochberg false discovery rate method by default. Genes were ordered according to the fold change in the expression values. This information was presented as a heat map and a volcano plot.

### 4.3. Data Processing of DEGs

GEO2R (https://www.ncbi.nlm.nih.gov/geo/geo2r/) was used to search DEGs between glioma samples and normal samples. GEO2R was an interactive online tool allowing users to compare two or more groups of samples in a GEO series and it analyzed most GEO series with gene symbol [38]. The adjusted *p* values were used to decrease the false positive rate using Benjamini and Hochberg false discovery rate method by default. The adjust *p* value < 0.01 and ∣logFC∣ ≥ 2 were set as the cutoff criterion. Then, 1980 DEGs were found, including 697 upregulated genes and 1283 downregulated genes, and the top 15 genes with a high degree of connectivity as hub genes were selected.

### 4.4. Gene Ontology and KEGG Pathway Analysis of DEGs

Gene ontology (GO) analysis served as a useful approach to annotate genes and gene products and also identify characteristic biological attributing to high-throughput genome or transcriptome data [39]. Kyoto Encyclopedia of Genes and Genomes (KEGG) was a collection of databases which helps to handle genomes, biological pathways, diseases, chemical substances, and drugs [40]. The Database for Annotation, Visualization and Integrated Discovery (DAVID, https://david.ncifcrf.gov/) was a web-based online bioinformatics resource that aims to provide tools for the functional interpretation of large lists of genes or proteins [41]. *p* < 0.05 was regarded as the cutoff criterion. We could visualize the core biological process (BP), molecular function (MF), cellular component (CC), and pathways among those DEGs using DAVID.

### 4.5. PPI Network and Module Analysis

Search Tool for the Retrieval of Interacting Genes (STRING) was a useful online tool designed to assess the protein-protein interaction (PPI) information [42]. To explore the potential relationship among those DEGs, we applied STRING app in Cytoscape and mapped the DEGs into STRING. And confidence score ≥ 0.4 and maximum number of interactors = 0 were set as the cutoff criterion. Moreover, the Molecular Complex Detection (MCODE) app was utilized to screen modules of the PPI network in Cytoscape with degree cutoff = 2, node score cutoff = 0.2, *k*-core = 2, and max. depth = 100. The pathway analysis of genes in these modules was performed by DAVID. In the meantime, 15 hub genes were also inserted into STRING with confidence score ≥ 0.4 and maximum number of interactors = 0. GO and KEGG pathway analyses were also carried out to identify the potential information.

### 4.6. Comparison of the Hub Gene Expression Level

The GEPIA (http://gepia.cancer-pku.cn/index.html) was a newly developed interactive web server that could analyze the RNA sequencing expression data of 9736 tumors and 8587 normal samples from the TCGA and the GTEx projects, using a standard processing pipeline [43]. It offered customizable functions involving tumor and normal differential expression analysis, and we could unveil the expression level of hub genes in glioma tissues and normal tissues. Then, the boxplots were performed to visualize the correlations. The Human Protein Atlas (HPA, https://www.proteinatlas.org/) was a Swedish-based program initiated in 2003 with the aim to map all the human proteins in cells, tissues, and organs using integration of various omics technologies, including antibody-based imaging, mass spectrometry-based proteomics, transcriptomics, and systems biology [44]. By acquiring immunohistochemical data of patients with or without glioma based on HPA, we further verified the expression of these hub genes.

### 4.7. Survival Analysis of Hub Genes

The relapse-free and overall survival information were based on TCGA database and the GTEx projects based on GEPIA [43]. The hazard ratio (HR) with 95% confidence intervals and log rank *p* value were calculated and displayed on the plot.

## Figures and Tables

**Table 1:** Top 15 hub genes with higher degree of connectivity.

Gene	Degree of connectivity	Adjusted *p* value
TP53	183	1.53*E* − 15
TOP2A	166	5.17*E* − 13
CDK1	146	3.55*E* − 17
PHLPP2	139	5.44*E* − 17
DLG4	124	4.68*E* − 18
CCNB1	115	3.09*E* − 05
MYC	115	2.43*E* − 04
CDC20	111	3.57*E* − 04
CCNA2	107	5.72*E* − 05
NDC80	105	2.00*E* − 10
AURKA	105	5.00*E* − 05
BIRC5	103	6.26*E* − 07
CCNB2	102	1.70*E* − 08
KIF11	102	5.41*E* − 11
MAD2L1	101	2.98*E* − 06

**Table 2:** Gene ontology analysis of differentially expressed genes associated with glioma.

Expression	Category	Term	Count	Ratio (%)	*p* value	FDR
Upregulated	GOTERM_BP_FAT	GO:0000278_mitotic cell cycle	105	15.21739	1.34*E* − 26	2.60*E* − 23
	GOTERM_BP_FAT	GO:1903047_mitotic cell cycle process	100	14.49275	1.81*E* − 26	3.51*E* − 23
	GOTERM_BP_FAT	GO:0022402_cell cycle process	120	17.3913	1.00*E* − 22	1.94*E* − 19
	GOTERM_BP_FAT	GO:0007067_mitotic nuclear division	62	8.985507	1.10*E* − 21	2.14*E* − 18
	GOTERM_BP_FAT	GO:0051301_cell division	71	10.28986	5.72*E* − 21	1.11*E* − 17
	GOTERM_MF_FAT	GO:0005201_extracellular matrix structural constituent	15	2.173913	2.78*E* − 07	4.37*E* − 04
	GOTERM_MF_FAT	GO:0015026_coreceptor activity	9	1.304348	2.11*E* − 05	3.32*E* − 02
	GOTERM_MF_FAT	GO:0017147_Wnt protein binding	8	1.15942	4.31*E* − 05	6.79*E* − 02
	GOTERM_MF_FAT	GO:0003777_microtubule motor activity	12	1.73913	4.41*E* − 05	6.93*E* − 02
	GOTERM_MF_FAT	GO:0019838_growth factor binding	15	2.173913	5.77*E* − 05	9.08*E* − 02
	GOTERM_CC_FAT	GO:0044420_extracellular matrix component	36	5.217391	1.74*E* − 21	2.56*E* − 18
	GOTERM_CC_FAT	GO:0005578_proteinaceous extracellular matrix	55	7.971014	3.38*E* − 19	4.99*E* − 16
	GOTERM_CC_FAT	GO:0031012_extracellular matrix	65	9.42029	1.05*E* − 17	1.54*E* − 14
	GOTERM_CC_FAT	GO:0005604_basement membrane	26	3.768116	3.08*E* − 15	4.59*E* − 12
	GOTERM_CC_FAT	GO:0015630_microtubule cytoskeleton	93	13.47826	2.04*E* − 14	3.01*E* − 11

Downregulated	GOTERM_BP_FAT	GO:0099536_synaptic signaling	194	15.39683	1.23*E* − 90	2.39*E* − 87
	GOTERM_BP_FAT	GO:0098916_anterograde trans-synaptic signaling	194	15.39683	1.23*E* − 90	2.39*E* − 87
	GOTERM_BP_FAT	GO:0099537_trans-synaptic signaling	194	15.39683	1.23*E* − 90	2.39*E* − 87
	GOTERM_BP_FAT	GO:0007268_chemical synaptic transmission	194	15.39683	1.23*E* − 90	2.39*E* − 87
	GOTERM_BP_FAT	GO:0007399_nervous system development	324	25.71429	2.19*E* − 60	4.25*E* − 57
	GOTERM_MF_FAT	GO:0022836_gated channel activity	95	7.539683	1.44*E* − 38	2.37*E* − 35
	GOTERM_MF_FAT	GO:0005216_ion channel activity	101	8.015873	2.22*E* − 33	3.67*E* − 30
	GOTERM_MF_FAT	GO:0022838_substrate-specific channel activity	101	8.015873	5.72*E* − 32	9.44*E* − 29
	GOTERM_MF_FAT	GO:0015267_channel activity	103	8.174603	1.31*E* − 30	2.16*E* − 27
	GOTERM_MF_FAT	GO:0022803_passive transmembrane transporter activity	103	8.174603	1.57*E* − 30	2.59*E* − 27
	GOTERM_CC_FAT	GO:0045202_synapse	232	18.4127	9.60*E* − 105	1.43*E* − 101
	GOTERM_CC_FAT	GO:0097458_neuron part	306	24.28571	1.50*E* − 104	2.23*E* − 101
	GOTERM_CC_FAT	GO:0044456_synapse part	202	16.03175	2.51*E* − 97	3.73*E* − 94
	GOTERM_CC_FAT	GO:0043005_neuron projection	232	18.4127	1.87*E* − 80	2.79*E* − 77
	GOTERM_CC_FAT	GO:0030424_axon	131	10.39683	4.44*E* − 58	6.61*E* − 55

GO: gene ontology; BP: biological process; MF: molecular function; CC: cell component; FDR: false discovery rate.

**Table 3:** KEGG pathway analysis of differentially expressed genes associated with glioma.

Expression	Term	Count	Ratio (%)	*p* value	Genes	FDR
Upregulated	hsa04110: cell cycle	23	3.333333	3.25*E* − 11	CDK1, E2F5, TP53, TTK, CHEK1, CDC20, CDK6, MCM2, SFN, CDK4, TGFB1, WEE1, CDK2, MCM5, CCNB1, CCND1, MAD2L1, CCNB2, CDKN2C, BUB1, BUB1B, MYC, CCNA2	4.06*E* − 08
	hsa04512: ECM-receptorinteraction	19	2.753623	1.55*E* − 10	TNC, COL3A1, HSPG2, ITGA2, ITGA4, COL4A6, COL5A1, HMMR, LAMA2, LAMA1, LAMA4, LAMB2, CD36, CD44, COL6A3, COL1A2, COL6A2, COL6A1, COL1A1	1.94*E* − 07
	hsa04115: p53 signaling pathway	17	2.463768	1.72*E* − 10	STEAP3, CDK1, TP53, CHEK1, CDK6, SFN, PMAIP1, CDK4, CDK2, CCNB1, TP53I3, CCND1, CCNB2, RRM2, CASP8, SERPINE1, FAS	2.16*E* − 07
	hsa05200: pathways in cancer	32	4.637681	2.06*E* − 06	WNT5A, STK36, ERBB2, MMP9, LPAR4, CXCL8, GLI2, TGFB1, TCF7L1, LAMB2, CXCR4, CASP8, FAS, MYC, BMP2, TGFBR1, TP53, ITGA2, BIRC5, CDK6, FZD2, CDK4, MECOM, COL4A6, CDK2, FZD6, LAMA2, SMO, LAMA1, LAMA4, CCND1, F2R	2.58*E* − 03
	hsa05161: hepatitis B	18	2.608696	3.02*E* − 06	TGFBR1, MMP9, TP53, HSPG2, CXCL8, TLR3, BIRC5, CDK6, CDK4, CDK2, TGFB1, CCND1, CASP8, CREB3L4, FAS, MYC, CCNA2, NFATC1	3.78*E* − 03

Downregulated	hsa04723: retrograde endocannabinoid signaling	38	3.015873	1.92*E* − 20	ADCY1, GABRB3, GABRB2, GABRB1, ADCY5, RIMS1, KCNJ3, SLC32A1, PLCB4, CNR1, MGLL, GNG3, PLCB1, GNG7, GABRD, GABRG1, GABRG2, GABRA2, GABRA1, GABRA4, GABRA5, PRKCG, GRIA3, GRIA4, GRM1, ITPR1, PRKCB, SLC17A7, GRM5, SLC17A6, KCNJ6, KCNJ9, GRIA2, FAAH, GNB5, CACNA1D, CACNA1A, CACNA1B	2.45*E* − 17
	hsa04724: glutamatergic synapse	40	3.174603	2.94*E* − 20	ADCY1, GRIK2, ADCY5, PPP3R1, GRIN3A, KCNJ3, SLC1A2, PLCB4, GRIN2C, SLC1A6, PPP3CB, DLG4, GNG3, PPP3CA, PLCB1, SLC1A1, GNG7, DLGAP1, GRIN2A, PRKCG, GRIA3, GRIA4, HOMER1, GRM1, SHANK2, SHANK3, ITPR1, PRKCB, GRM5, SLC17A7, GRM3, GRM2, SLC17A6, GRIA2, GRM7, GNB5, PLA2G4C, CACNA1D, GRK3, CACNA1A	3.76*E* − 17
	hsa05032: morphine addiction	32	2.539683	2.87*E* − 16	ADCY1, GABRB3, GABRB2, ADCY5, GABRB1, GABBR1, GABBR2, ADORA1, KCNJ3, SLC32A1, PDE1C, PDE4A, PDE1A, GNG3, GNG7, GABRG1, GABRD, GABRG2, GABRA2, GABRA1, GABRA4, GABRA5, PRKCG, PDE10A, PRKCB, KCNJ6, PDE2A, KCNJ9, GNB5, GRK3, CACNA1A, CACNA1B	4.22*E* − 13
	hsa04727: GABAergic synapse	30	2.380952	2.91*E* − 15	ADCY1, SLC6A1, GABRB3, GABRB2, ADCY5, GABRB1, GABBR1, GABBR2, SLC32A1, PLCL1, GAD2, GNG3, GAD1, NSF, GNG7, GABRG1, GABRD, GABRG2, GABARAPL1, GABRA2, GABRA1, GABRA4, GABRA5, PRKCG, PRKCB, KCNJ6, GNB5, CACNA1D, CACNA1A, CACNA1B	3.69*E* − 12
	hsa04020: calcium signaling pathway	43	3.412698	5.62*E* − 15	SLC8A3, ADCY1, ERBB3, CAMK2G, PPP3R1, ITPKA, ATP2B2, ATP2B3, PLCB4, GRIN2C, PDE1C, PTK2B, PDE1A, PPP3CB, CAMK2B, PPP3CA, PLCB1, CAMK2A, NOS1, SLC8A2, CCKBR, CACNA1I, GRIN2A, PRKCG, GRM1, ITPR1, PRKCB, P2RX5, GRM5, GNAL, ADRB1, CAMK4, CHRM3, CHRM1, CACNA1G, RYR1, RYR2, CACNA1E, CACNA1D, HTR2C, CACNA1A, CACNA1B, HTR2A	7.24*E* − 12

KEGG: Kyoto Encyclopedia of Genes and Genomes; FDR: false discovery rate.

**Table 4:** The enriched pathways of top 4 modules from the protein-protein interaction network.

Module	Term	*p* value	FDR	Genes
1	Cell cycle	7.17*E* − 13	5.94*E* − 10	CCNB1, CDK1, MAD2L1, CCNB2, BUB1, TTK, BUB1B, CHEK1, CDC20, MCM2, CCNA2
	Progesterone-mediated oocyte maturation	4.89*E* − 06	4.05*E* − 03	CCNB1, CDK1, MAD2L1, CCNB2, BUB1, CCNA2
	p53 signaling pathway	4.27*E* − 05	3.54*E* − 02	CCNB1, CDK1, CCNB2, RRM2, CHEK1
	Oocyte meiosis	2.85*E* − 04	0.235961	CDK1, MAD2L1, BUB1, AURKA, CDC20
	DNA replication	5.21*E* − 03	4.233590	POLE2, MCM2, RNASEH2A

2	Neuroactive ligand-receptorinteraction	4.44*E* − 11	4.86*E* − 08	OPRK1, GABBR1, NPY1R, GABBR2, ADORA1, S1PR3, GRM3, SSTR2, GRM2, GRM7, CNR1, S1PR5, ADRA2A
	Morphine addiction	2.16*E* − 08	2.36*E* − 05	ADCY1, ADCY5, GABBR1, GNB5, GABBR2, GNG3, ADORA1, GNG7
	Glutamatergic synapse	1.04*E* − 07	1.14*E* − 04	ADCY1, GRM3, GRM2, ADCY5, GRM7, GNB5, GNG3, GNG7
	GABAergic synapse	4.26*E* − 07	4.66*E* − 04	ADCY1, ADCY5, GABBR1, GNB5, GABBR2, GNG3, GNG7
	cAMP signaling pathway	4.42*E* − 06	4.84*E* − 03	ADCY1, SSTR2, NPY, ADCY5, GABBR1, GABBR2, NPY1R, ADORA1

3	Calcium signaling pathway	2.75*E* − 08	2.95*E* − 05	GRM5, PLCB4, CCKBR, CHRM3, CHRM1, PLCB1, GRM1, HTR2C, HTR2A, F2R
	Neuroactive ligand-receptorinteraction	9.29*E* − 08	9.95*E* − 05	GRM5, CCKBR, CHRM3, CHRM1, F2RL1, LPAR4, NTSR2, GRM1, HTR2C, HTR2A, F2R
	Gap junction	2.79*E* − 05	2.99*E* − 02	GRM5, PLCB4, PLCB1, GRM1, HTR2C, HTR2A
	Endocytosis	7.19*E* − 05	7.69*E* − 02	SH3GL3, DNM3, DAB2, DNAJC6, DNM1, SH3GL2, F2R, AMPH
	Inflammatory mediator regulation of TRP channels	6.88*E* − 04	0.734597	PLCB4, F2RL1, PLCB1, HTR2C, HTR2A

4	Arrhythmogenic right ventricular cardiomyopathy (ARVC)	3.06*E* − 12	3.25*E* − 09	CACNA2D1, CACNG8, CACNB2, RYR2, CACNG3, CACNB3, CACNG2, CACNB4, CACNA2D3, CACNA1D
	Cardiac muscle contraction	5.12*E* − 12	5.43*E* − 09	CACNA2D1, CACNG8, CACNB2, RYR2, CACNG3, CACNB3, CACNG2, CACNB4, CACNA2D3, CACNA1D
	Hypertrophic cardiomyopathy (HCM)	7.38*E* − 12	7.82*E* − 09	CACNA2D1, CACNG8, CACNB2, RYR2, CACNG3, CACNB3, CACNG2, CACNB4, CACNA2D3, CACNA1D
	Dilated cardiomyopathy	1.47*E* − 11	1.56*E* − 08	CACNA2D1, CACNG8, CACNB2, RYR2, CACNG3, CACNB3, CACNG2, CACNB4, CACNA2D3, CACNA1D
	Oxytocin signaling pathway	1.77*E* − 10	1.88*E* − 07	CACNA2D1, CCND1, CACNG8, CACNB2, RYR2, CACNG3, CACNB3, CACNG2, CACNB4, CACNA2D3, CACNA1D

FDR: false discovery rate.

## Data Availability

The data used to support the findings of this study are available from the corresponding author upon request.
